# Deterministic error correction for nonlocal spatial-polarization hyperentanglement

**DOI:** 10.1038/srep20677

**Published:** 2016-02-10

**Authors:** Tao Li, Guan-Yu Wang, Fu-Guo Deng, Gui-Lu Long

**Affiliations:** 1 State Key Laboratory of Low-Dimensional Quantum Physics and Department of Physics, Tsinghua University, Beijing 100084, China; 2 Department of Physics, Applied Optics Beijing Area Major Laboratory, Beijing Normal University, Beijing 100875, China; 3 Tsinghua National Laboratory of Information Science and Technology, Beijing 100084, China; 4 Collaborative Innovation Center of Quantum Matter, Beijing 100084, China

## Abstract

Hyperentanglement is an effective quantum source for quantum communication network due to its high capacity, low loss rate, and its unusual character in teleportation of quantum particle fully. Here we present a deterministic error-correction scheme for nonlocal spatial-polarization hyperentangled photon pairs over collective-noise channels. In our scheme, the spatial-polarization hyperentanglement is first encoded into a spatial-defined time-bin entanglement with identical polarization before it is transmitted over collective-noise channels, which leads to the error rejection of the spatial entanglement during the transmission. The polarization noise affecting the polarization entanglement can be corrected with a proper one-step decoding procedure. The two parties in quantum communication can, in principle, obtain a nonlocal maximally entangled spatial-polarization hyperentanglement in a deterministic way, which makes our protocol more convenient than others in long-distance quantum communication.

Quantum entanglement is an important resource for quantum computation and quantum communication. The ability of entanglement creation between distant locations is usually a prerequisite for quantum communication, such as quantum teleportation^1^, quantum dense coding (QDC)^2^,^3^, quantum key distribution^4^,^5^, quantum secret sharing^6^, quantum secure direct communication^7^,^8^,^9^,^10^, and quantum imaging^11^. However, the direct distribution of quantum entanglement over an optical-fiber channel without any protection will inevitably make the entanglement suffer from the channel noise, which results in its degradation^4^,^12^. To mitigate the channel noise and implement quantum communication securely between distant parties, several useful methods have been presented^13^,^14^,^15^,^16^,^17^,^18^,^19^, such as quantum error correct code (QECC)^13^, quantum error-rejection code (QERC)^14^,^15^, and decoherence free subspace (DFS)^16^,^17^,^18^,^19^. The ideal redundant encoding of QECC is essentially the same as the classical one, where one logical qubit is encoded by several physical qubits according to the type of the noise, and the correction procedure can be performed after error detecting^13^. The QERC and DFS are two effective methods to suppress the collective error noise during the photon propagation^14^,^15^,^16^,^17^,^18^,^19^. By picking out the photon in a deterministic time slot, both the two-photon QERC and the single-photon QERC can correct the polarization noise during the photon propagation with proper encoding and decoding procedures, and provide the parties a faithful channel with two fiber channels in a probabilistic way^14^,^15^.

Entanglement purification is an important passive way to improve the fidelity of nonlocal entangled quantum systems polluted by the quantum channel noise, and it is of vital importance when it is combined with quantum entanglement swapping to implement the quantum repeater network^20^,^21^,^22^. The first entanglement purification protocol (EPP) was proposed in 1996 by Bennett *et al*.^23^ with controlled-not (CNOT) gates for the nonlocal quantum systems entangled in one degree of freedom (DOF). Subsequently, Bennett *et al*.^24^ showed that EPP is deeply connected to QECC, especially that the EPP involving classical communication is in principle equivalent to QECC. In the EPP, the two-qubit operations, i.e., CNOT operation or parity-check operation are exploited to detect the errors and the feedback operations along with the measurement on the auxiliary systems result in the error correction. In 2001, Pan *et al*.^25^ introduced a convenient EPP for polarization entanglement from an ideal entanglement source resorting to linear optical elements. In 2002, Simon and Pan^26^ developed an EPP with linear optical elements for the currently available parametric down-conversion source, and took the spatial entanglement as a resource to purify the polarization entanglement when the bit-flip error correction was needed^27^. In 2008, Sheng *et al*.^28^ developed an EPP using deterministic quantum non-demolition detection (QND) with cross-Kerr nonlinearity and it could be used to implement multi-step purification for mixed polarized entangled systems. In 2014, Li, Yang, and Deng^29^ designed a high-efficiency EPP for atomic ensembles. The EPPs described above can be considered as a hierarchical error correction for the polarization errors, and they consume largely the less entangled quantum systems.

Entanglement purification^23^,^24^,^25^,^26^,^27^,^28^,^29^,^30^ makes great progress when the concept of deterministic entanglement purification was introduced originally by Sheng and Deng^31^ in 2010. The polarization errors can be corrected with the error-free entanglement of the photon pair itself in another one or two DOFs, and each photon pair purified is in the maximally entangled state in the polarization DOF with a unity efficiency. In the first deterministic entanglement purification protocol (DEPP)^31^, the hyperentanglement^32^ in both the spatial DOF and the frequency DOF was exploited to purify the polarization entanglement and a two-step error correction for the bit-flip error and the phase-flip error was presented. Subsequently, Sheng *et al*.^33^ and Li^34^ proposed two similar one-step DEPPs independently with linear optical elements. The polarization errors are deterministically converted into the ambiguity of the spatial modes of the photons when the proper feedback operations are involved. In 2011, Deng^35^ introduced a general one-step error correction for multipartite polarization entanglement and pointed out the equivalence between the auxiliary spatial entanglement and frequency entanglement. Moreover, he showed that a DEPP does not require the photon systems entangled in the polarization DOF, but one error-free DOF. These DEPPs can purify the polarization entanglement with one step and the polarization errors are totally converted into the ambiguity of spatial modes when the two photons in each pair are originally entangled in spatial DOF which has been exploited to generation a (100 × 100)-dimensional entanglement^36^. Compared with the conventional EPPs^23^,^25^,^26^,^27^,^28^,^29^, these one-step DEPPs^33^,^34^,^35^ reduce the requirement on the interference coherence in time of the two photons from two pairs, which makes them feasible in practical quantum repeaters. Recently, some interesting DEPPs are presented, such as the DEPP with the time-bin entanglement^37^ and the DEPP resorting to QND and spatial entanglement^38^, which is used to perform a secure double-server blind quantum computation. Besides, Wang *et al*.^39^ introduced a heralded hyperdistillation scheme with linear optical elements. Zhou and Sheng presented a recyclable amplification protocol^40^ for the single-photon entangled state assisted by QND, which could increase the success probability of the single photon transmission and be quite useful in quantum network.

Hyperentanglement, the entanglement in multiple DOFs of a quantum system^41^,^42^,^43^,^44^, has attracted much attention as it has some important applications in quantum communication, such as hyperentanglement-assisted linear Bell-state analyzing^45^, the “All-Versus-Nothing” proof of Bell's theorem^46^, the efficient QDC^47^,^48^, hyperentangled Bell-state analysis^49^,^50^,^51^,^52^, the quantum teleportation of multiple DOFs of a quantum particle^53^, and hyperentanglement swapping^50^,^51^. Hyperentanglement purification attracts much attention recently, and some interesting hyperentanglement purification protocols (HEPP) are introduced. In 2013, Ren *et al*.^54^ firstly presented an HEPP for spatial-polarization hyperentangled photonic systems with two individual quantum non-demolition parity-check detectors for both the spatial DOF and the polarization DOF assisted by diamond nitrogen-vacancy centers embedded in photonic crystal microcavities. Subsequently, Ren and Deng^55^ proposed a high-efficiency two-step HEPP with quantum-state joining method, and the original states discarded with a poorer fidelity in either the spatial DOF or the polarization DOF were recycled to perform the quantum-state joining procedure, leading to a high efficiency. Recently, Wang *et al*.^56^ introduced a one-step HEPP with linear optics. The quantum non-demolition parity-check measurements for the spatial DOF and the polarization DOF could be completed simultaneously in a heralded way when local multi-photon entanglement was available^57^.

In this paper, we propose an efficient deterministic error correction for spatial-polarization hyperentanglement of nonlocal photonic systems assisted by the time-bin entanglement^58^,^59^. When the maximal time delay between each two time-bins is small, compared to the variation of the fluctuation and birefringence of the fiber, the photons in each time-bin will suffer an identical polarization noise, which is referred to as the collective error model^14^,^15^,^16^,^17^,^18^,^19^. The parties in quantum communication first encode the spatial-polarization hyperentanglement into spatially defined time-bin entanglement. When the photons propagate along the fiber channels, the fluctuation of fiber only results in a global phase and the noise leading to spatial errors can be rejected. Meanwhile, the birefringence of the fiber will inevitably introduce a polarization error, i.e., the bit-flip error or the phase-flip error^14^,^15^,^16^,^17^,^18^,^19^. The parties finally perform a faithful one-step decoding procedure to complete the error correction, and they can obtain a maximal spatial-polarization hyperentanglement of photonic systems as the hyperentanglement concentration does^60^,^61^,^62^,^63^. Moreover, our error-correction protocol works in a deterministic way, the same as the DEPPs for the polarization entanglement only^31^,^33^,^34^,^35^,^37^,^38^, and the success probability is 100% in principle. The protocol is based on linear optical elements and the well developed fast Pockels cells (PCs) or widely used fiber polarization controller^64^. It is, therefore, in reach of experimental implementation and it has good applications in the practical quantum network.

## Results

### Time-bin encoding and the collective noise channel

Suppose there is a hyperentanglement source located at the middle of two parties in quantum communication, say Alice and Bob, and it generates photon pairs hyperentangled in both the spatial and polarization DOFs^41^,^42^,^43^:


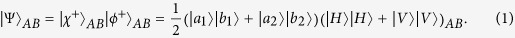


Here the subscripts *A* and *B* are used to discriminate the photons sent to the two remote parties in quantum communication, say Alice and Bob, respectively. 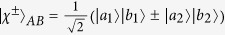 are used to denote the spatial Bell states of the photon pair *AB*. 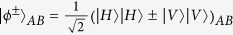 and 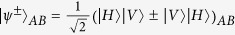 are used to denote their polarized Bell states. *H* and *V* represent the horizontal and vertical polarizations of photons, respectively. *a*_1_ and *a*_2_ (*b*_1_ and *b*_2_) represent the two spatial modes of the photon *A* (*B*) sent to Alice (Bob).

**Figure 1:**
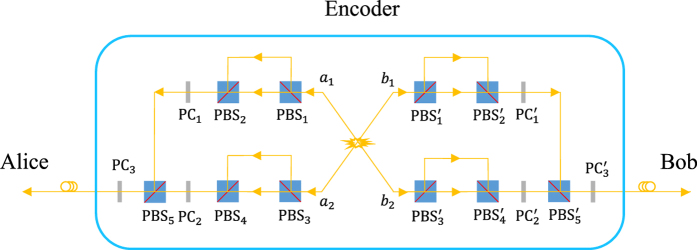
Schematic diagram for the time-bin entanglement encoding in nonlocal spatial-polarization hyperentanglement distribution. PBS_*i*_ 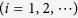 represents a polarizing beam splitter that transmits the horizonal photons 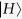 and reflects the vertical polarized photons 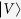. PC_*i*_ stands for a Pockels cell which completes a polarization bit-flip operation 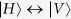 on the photon passing through when it is activated.

Before launching the photons *AB* into the fiber channels, the parties perform a time-bin encoding procedure and transform the spatial-polarization hyperentanglement into a spatial defined time-bin entanglement with 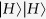 polarization using our encoder shown in Fig. 1. This encoder is composed of some unbalanced polarizing interferometers and PCs which implement the bit-flip operation 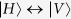 when they are activated. It will introduce a time delay *t* on the 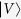 component of the photon when it passes through an interferometer, since 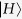 is transmitted by the polarizing beam splitter (PBS) and propagates along the short arm, while 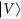 is reflected by the PBS and passes through the long arm. After the photons passes through the unbalanced polarizing interferometers, the parties apply the time-dependent polarization-flip operations to make the upper modes *a*_1_ and *b*_1_ in 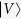 polarization and the down modes *a*_2_ and *b*_2_ in 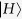 polarization, i.e., effecting the transformation 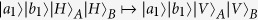 and 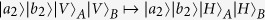. Besides, an optical delay *t*′ much longer than the time delay *t* introduced by the interferometers is applied on the 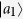 and 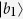 spatial modes. The state of the photons *AB* evolves into


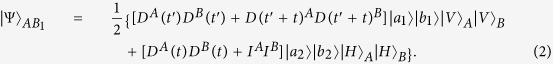


Here *D*^*i*^(*t*) 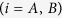 is a linear time-delay operator which implements a time delay on the photon *i*, and it satisfies the following relations:


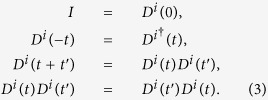


Now, the polarization DOF of the photons *AB* are entangled with their spatial modes, and time-bins with different time delays can be used to discriminate the particular spatial and polarization components of the original hyperentanglement when the spatial and polarization information has been erased.

To get the spatial defined time-bin entanglement with the polarization 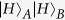, the *a*_1_ and *a*_2_ (*b*_1_ and *b*_2_) components are combined together at *PBS*_5_ 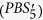 and the 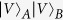 polarized components labeled with the time delay 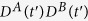 or 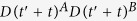 can be transformed into 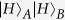 with some polarization-flip operations at the corresponding time slots. The photons launched into the fiber channels are in the state:


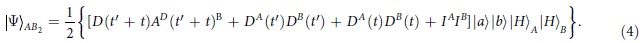


Notice that the dominant noise affecting the photons propagating through fiber channels is the collective noise as long as the photons travel inside a small time window, compared to the fluctuation of the optical path and the variation of the birefringence, the transformations on each time-bin component of a single photon are also the same as each other^14^,^15^,^16^,^17^,^18^,^19^. After a pair of photons *AB* pass through the noise channel, up to a global phase, the state of the photons is then described as:


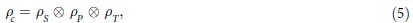


where 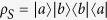 represents the spatial mode, 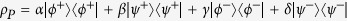 with 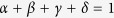 denotes the polarization mode, 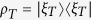 with 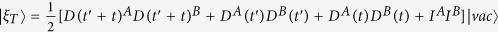 is the time-bin mode, and the vacuum state 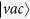 corresponds to the time-bin one without any time delay.

In a different notation, the two photons *AB* in the mixed state *ρ*_*c*_ are composed of four pure states and could be written as


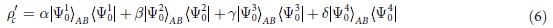


with


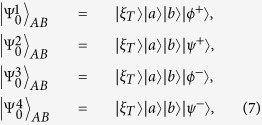


and the corresponding probabilities that photons *AB* are projected into 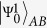, 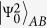, 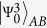, and 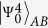 are *α, β, γ*, and *δ*, respectively. One can easily find that there are three kinds of errors in the polarization DOF of the mixed state 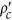, i.e., the bit-flip error case 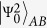, the phase-flip error case 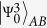, and the case with both phase-flip and bit-flip errors 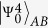. In the following section, we will detail how the distant parties can obtain the error-free photon pairs maximally hyperentangled in both spatial DOF and polarization DOF from the mixed state 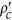 shown in Eq. (6). No matter which types of errors take place, the parties can perform the error correction procedure in a deterministic way and transfer the polarization error into time-bin ambiguity in the one-step error-correction decoding procedure, leaving the photon pairs in the state 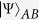 that is maximally entangled in both spatial DOF and polarization DOF.

### One-step error-correction decoding for nonlocal spatial-polarization hyperentanglement

**Figure 2:**
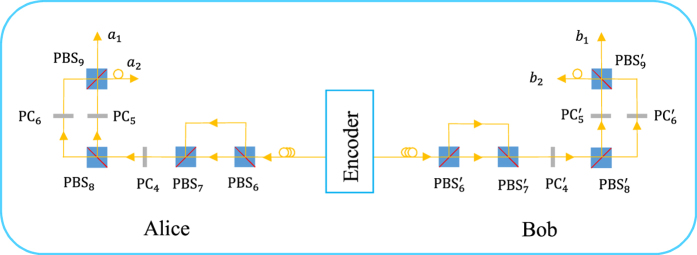
Schematic diagram for error correction decoding in nonlocal spatial-polarization hyperentanglement distribution. Encoder represents the time-bin encoding setup that transforms the spatial-polarization hyperentanglement into the time-bin entanglement.

The principle of our one-step error-correction decoding procedure for nonlocal hyperentanglement distribution is shown in Fig. 2. Each decoder is made up of unbalanced interferometers and PCs. To demonstrate the validity and reliability of the decoding procedure, we first discuss the case that photons *AB* are in the state 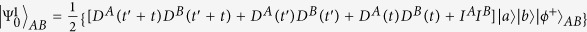, and then we will detail what the decoding procedure for 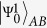 will do for the case 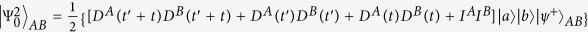 where a bit-flip error takes place, and give out the photonic states evolving with the same decoding procedure when the other two types of errors take place.

For the case that photons *AB* are in the state 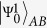, after passing through the first unbalanced interferometer made up of *PBS*_6_ and *PBS*_7_ (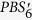 and 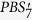), the photon *A* (*B*) with the vertical polarization 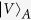 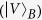 is delayed by an optical delay *t* represented by applying an operator 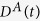 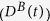 on it. The state of *AB* evolves into


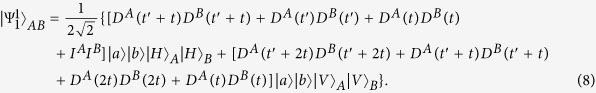


Second, the parties perform a bit-flip operation on the polarization DOF of *AB* when they arrive at the time slots with the time delay *t* or *t* + *t*′. And then, the parties introduce an optical delay *t* on the photon *A* (*B*) with the horizonal polarization 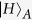 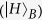 by using a longer optical path in the transmission mode of *PBS*_8_ 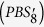. The state of the photons *AB* is transformed into a polarization-time-bin hyperentangled one


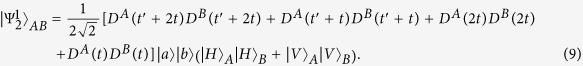


By far, the spatial DOF of *AB* is kept in the product state 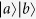 and it does not suffer from the collective noise. To recover the spatial-polarization hyperentanglement, the parties activate the polarization-flip operations when the scheduled photons with the time delays *t*′ + *t* and *t*′ + 2*t* arrive. The photons at the outputs of *PBS*_9_ and 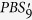 are in a hybrid spatial-polarization-time-bin hyperentangled state that can be described as:


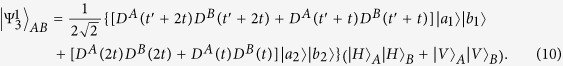


To disentangle spatial-time-bin entanglement in the state 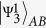 and remove the time-bin difference on the spatial modes *a*_1_*b*_1_ and *a*_2_*b*_2_, one can apply an optical delay of the scale *t*′ on the spatial modes *a*_2_*b*_2_ with the delay operator 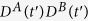, and the final photon pair *AB* shared by Alice and Bob is in the spatial-polarization hyperentangled state:


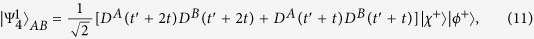


where the photons can occupy the time slot with the optical delay 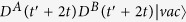 or 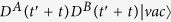.

For the case that a bit-flip error takes place on the polarization entangled DOF 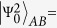 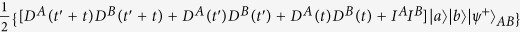, with the same decoding procedure as that discussed above, the two parties in quantum communication will also obtain the maximally spatial-polarization hyperentangled state. The evolution of the photon pair system in this situation can be detailed in a similar way.

First, with the optical delay *t* on the vertical polarized component 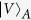 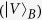 shown in Fig. 2, 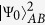 is projected into


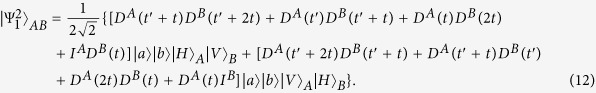


Second, Alice and Bob complete the bit-flip operation 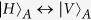 and 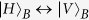 on the components with the scheduled delay *t* or *t* + *t*′ of *A* and *B*, respectively. And then, with the optical delay *t* on 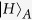 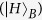, the photon system will also be transformed into a polarization-time-bin hyperentanglement of the following form:


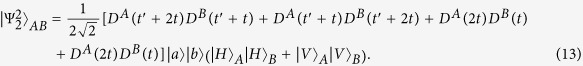


The photons are then scheduled to suffer from the bit-flip operation on the polarization DOF when they are in the components of the time delay *t*′ + *t* or *t*′ + 2*t*. The photons *AB* emitting from *PBS*_9_ and 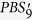 evolve into another hybrid spatial-polarization-time-bin hyperentangled state:


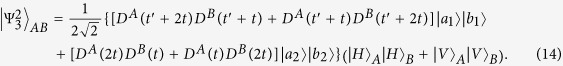


Finally, with the optical delay of *t*′ on the spatial modes *a*_2_*b*_2_ with the delay operator 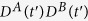, the two parties will also disentangle the spatial-time-bin entanglement in the state 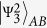 and project the photon pair *AB* into the desired spatial-polarization hyperentangled state:


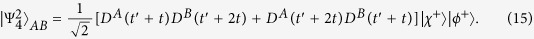


Here the time-bin slots that the parties can obtain the spatial-polarization hyperentanglement are different for the photon *A* and the photon *B* in 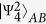, and it can be viewed as a bit-flip error on the time-bin entangled DOF, compared with that in 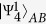.

As for the other two cases with the phase-flip error 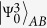, and both the phase-flip error and bit-flip error 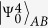, the parties will also obtain the desired spatial-polarization hyperentangled state 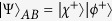 by using our error-correction decoding procedure described above, and the final hyperentangled states taking the time-bin information into account for the cases 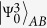 and 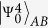 are, respectively,


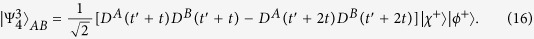


and


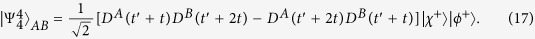


The corresponding polarization errors are simultaneously corrected faithfully, since they are totally transferred into the time-bin errors of the photons *AB*. No matter what time-bins the photons occupy, they are all in the desired spatial-polarization hyperentangled state 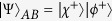.

## Discussion

By far, we have detailed our deterministic error correction protocol for nonlocal spatial-polarization hyperentanglement distribution of two-photon systems. Our method relies on the spatial-polarization and time-bin encoding of photons. Recent experiments have demonstrated that the time-bin encoding is a particularly robust photon qubit under the collective noise channels when the fiber channels are involved^65^,^66^,^67^,^68^. The distribution of time-bin entanglement at the scale of 50 km optical fiber was implemented and the photons after propagation still violate the Clauser-Horne-Shimony-Holt Bell inequality by more than 15 standard deviations without removing the detector noise^69^. Recently, the faithful distribution of time-bin entangled photon pairs over 300 km of fiber has been implemented^70^ and it confirms the reliability of the time-bin encoding of photon qubits^14^,^15^. Therefore, with the time-bin encoding, the parties in quantum communication can complete a spatial error-rejecting hyperentanglement distribution, while the polarization error can be completely corrected with a simple one-step error-correction decoding procedure.

Different from the interesting HEPPs^54^,^55^,^56^,^57^ in which the parties in quantum communication depress the noise in a passive way, our positive deterministic error correction for the hyperentangled photons works in an active way for collective noise and it enjoys the following advantages. The polarization errors, in our protocol, can be corrected in a deterministic way, since they are completely transferred into the time-bin errors leaving the spatial mode an additional DOF for photon qubit encoding. We can perform a deterministic error correction for the spatial-polarization entanglement of photon systems after they are transmitted over the noisy channels. The non-demolition parity-checking measurements as a result of cavity quantum electrodynamics or the interference assisted by local entanglement in the previous HEPPs are unnecessary in the present protocol. In other words, we can in principle distil a pair of maximally spatial-polarization hyperentangled photons from a single hyperentangled photon pair polluted by the noisy channels. Besides, our protocol does not consume largely the less-entangled systems as that in the positive entanglement distillation protocols, where the fidelity of the less-entangled systems are improved by repeating the purification procedures^23^,^24^,^25^,^26^,^27^,^28^.

The deterministic performance of our scheme comes from the conversion between the errors in polarization DOF and the ambiguity of the time-bin DOF. Fortunately, the minor time-bin ambiguity in the spatial-polarization hyperentangled photons can be eliminated automatically when performing entanglement swapping with the hyperentangled Bell-state analysis based on cavity quantum electrodynamics^50^,^51^,^52^. Since the scale of the time-bin ambiguity of the photons is smaller than the coherence time of the assisted stationary qubit, i.e., quantum dot (QD)^50^,^51^ or NV centers^52^, used to perform the swapping procedure, and only the spatial DOF and polarization DOF of the photons will affect the stable photonic output state of the cavity containing the stationary qubit^71^,^72^,^73^. The outcomes of the photon detection will herald the success of the swapping procedure and can project the remaining two photons in the desired spatial-polarization hyperentangled state 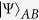 up to some local single qubit operations^50^,^51^,^52^. Meanwhile, the time-bin ambiguity of the photons can also be eliminated by the quantum memory devices involved in the quantum repeater network^74^,^75^,^76^,^77^.

In summary, we have presented an efficient deterministic error-correction protocol for spatial-polarization hyperentanglement distribution over collective-noise channels. By exploiting the robust time-bin entanglement, the parties in quantum communication can obtain a maximal spatial-polarization hyperentanglement. The polarization errors are totally converted into the time-bin errors, while the spatial errors can be rejected inherently as only one fiber channel is involved for each photon. Combined with the hyperentangled-Bell-state analysis^49^,^50^,^51^,^52^,^53^ and the quantum memory for hyperentangled photon pairs^74^,^75^,^76^,^77^, the present protocol can be utilized to perform a high-efficiency quantum repeater of multiple DOFs^78^. It can also be easily extended to the case for the multipartite hyperentanglement, and it will constitute an important building block for quantum communication and computation networks.

## Additional Information

**How to cite this article**: Li, T. *et al*. Deterministic error correction for nonlocal spatial-polarization hyperentanglement. *Sci. Rep.* **6**, 20677; doi: 10.1038/srep20677 (2016).
